# Modulation by Ozone of Glucocorticoid-Regulating Factors in the Lungs in Relation to Stress Axis Reactivity

**DOI:** 10.3390/toxics9110290

**Published:** 2021-11-03

**Authors:** Jith Thomas, Errol M. Thomson

**Affiliations:** 1 Environmental Health Science and Research Bureau, Healthy Environments and Consumer Safety Branch, Health Canada, Ottawa, ON K1A 0K9, Canada; jith.thomas@canada.ca; 2 Department of Biochemistry, Microbiology, and Immunology, Faculty of Medicine, University of Ottawa, Ottawa, ON K1H 8M5, Canada

**Keywords:** ozone, glucocorticoid, glucocorticoid metabolism, corticosteroid-binding globulin, elastase, inflammation, susceptibility

## Abstract

Exposure to air pollutants increases levels of circulating glucocorticoid stress hormones that exert profound effects relevant to health and disease. However, the nature and magnitude of tissue-level effects are modulated by factors that regulate local glucocorticoid activity; accordingly, inter-individual differences could contribute to susceptibility. In the present study, we characterized effects of ozone (O_3_) inhalation on glucocorticoid-regulating factors in the lungs of rat strains with contrasting hypothalamic–pituitary–adrenal stress axis responses. Hyper-responsive Fischer (F344) and less responsive Lewis (LEW) rats were exposed to air or 0.8 ppm O_3_ for 4 h by nose-only inhalation. Levels of the high-specificity and -affinity corticosteroid-binding globulin protein increased in the lungs of both strains proportional to the rise in corticosterone levels following O_3_ exposure. Ozone reduced the ratio of 11β-hydroxysteroid dehydrogenase type 1 (HSDB1)/HSDB2 mRNA in the lungs of F344 but not LEW, indicating strain-specific transcriptional regulation of the major glucocorticoid metabolism factors that control tissue-level action. Intercellular adhesion molecule (ICAM)-1 and total elastase activity were increased by O_3_ in both strains, consistent with extravasation and tissue remodeling processes following injury. However, mRNA levels of inflammatory markers were significantly higher in the lungs of O_3_-exposed LEW compared to F344. The data show that strain differences in the glucocorticoid response to O_3_ are accompanied by corresponding changes in regulatory factors, and that these effects are collectively associated with a differential inflammatory response to O_3_. Innate differences in glucocorticoid regulatory factors may modulate the pulmonary effects of inhaled pollutants, thereby contributing to differential susceptibility.

## 1. Introduction

A growing number of studies have demonstrated that health effects of air pollutants vary significantly within the population; however, mechanisms underlying inter-individual differences in susceptibility remain unclear. Glucocorticoids are major stress hormones produced by the adrenal cortex under regulation of the hypothalamic-pituitary adrenal (HPA) axis [1,2]. Normal adrenal cortical function is essential to resolve systemic and local inflammation, and dysfunction leads to various pathophysiological conditions [3,4]. Acute exposure to air pollutants (ozone [O_3_], particulate matter, diesel exhaust) has been demonstrated to increase concentrations of plasma glucocorticoids (corticosterone in rodents, cortisol in humans) [5,6,7,8]. We have shown previously that pharmacological inhibition of glucocorticoid synthesis increased O_3_-induced lung inflammatory responses, suggesting that glucocorticoids play a key role in regulating air pollutant-induced lung inflammatory responses [9]. Moreover, innate differences in stress axis function are associated with differential glucocorticoid and lung inflammatory response to O_3_ [10].

Although such studies have shown a relationship between circulating glucocorticoid levels and effects in the lungs, it is clear that local factors are critical regulators of glucocorticoid action [11,12,13]. Major factors that alter glucocorticoid availability in the lungs include binding proteins (e.g., corticosteroid -binding globulin [CBG] and albumin), glucocorticoid receptors, and/or 11β-hydroxysteroid dehydrogenase (HSDB)1 and HSDB2 enzymes [11,12,14]. While a number of studies have demonstrated that endogenous glucocorticoids modify lung inflammatory responses [15,16], less attention has been given to local factors in the lungs that may modify glucocorticoid effects.

The present study aimed to investigate how O_3_ inhalation regulates factors that control glucocorticoid activity in the lungs, and how inter-individual differences in response are reflected in differences in O_3_-induced inflammatory responses. Fischer 344 (F344) rats have a hyper-responsive stress axis, whereas Lewis (LEW) rats tend to be hypo-responsive [17,18]. In turn, LEW have been found to be susceptible to inflammatory diseases, whereas F344 exhibit resilience [19,20]. Moreover, F344- and LEW-like phenotypes are reported in humans [21,22,23], making them a useful model for investigating modifying factors of glucocorticoid responses in lungs and how these factors contribute to health effects of O_3_.

## 2. Material and Methods

### 2.1. Experimental Model

All experimental methods were reviewed and approved by the Animal Care Committee of Health Canada (Identification code: ACC#2017-004; Date of approval: 8 February 2017), and adhered to the Canadian Council on Animal Care guidelines for humane animal use. Details on housing and experimental procedures were described previously [10]. Briefly, male F344 and LEW rats (~250–300 g) were purchased from Charles River (St. Constant, QC, Canada), and progressively trained in nose-only exposure tubes over 7 consecutive days for acclimatization prior to the study. Nose-only exposures to clean air or 0.8 ppm O_3_ (800 ± 13 ppb) were performed for 4 h (*n* = 5/group), followed by anesthetization by isoflurane (5% at 1.5 L of O_2_/min) and euthanizing by exsanguination immediately at the end of the exposure.

### 2.2. Biological Samples

The lungs were washed-out with warm saline (37 °C) at 30 mL/kg body weight to obtain bronchoalveolar lavage fluid (BALF). Lavage samples were centrifuged (491× *g* for 10 min at 8 °C) to pellet cells, and the supernatant was frozen at −80 °C for subsequent analyses. The lungs were excised and frozen at −80 °C for total RNA extraction and transcript analysis.

### 2.3. Lavage Corticosteroid-Binding Globulin and Soluble Intracellular Adhesion Molecule-1 (ICAM-1)

Lavage corticosteroid-binding globulin and ICAM-1 were quantified using commercially available immunoassay kits (MyBioSource Rat Corticosteroid-binding globulin ELISA Kit, San Diego, CA, USA and Invitrogen ICAM-1 Rat ELISA Kit; Thermo Fisher Scientific, Waltham, MA, USA) according to the manufacturer’s protocols.

### 2.4. Total Elastase Activity

Total elastase activity in BALF was measured using an elastase activity assay kit (Cayman Chemicals, Ann Arbor, MI, USA). Briefly, elastase activity was measured using a substrate (Z-Ala-Ala-Ala-Ala 2Rh110) and production of the highly fluorescent product (compound R110) was measured using 485 nm excitation and 525 nm emission wavelengths in a fluorescence plate reader (SpectraMax^®^, Molecular Devices, San Jose, CA, USA).

### 2.5. RNA Extraction

RNA extraction of lung tissue samples was performed using Trizol reagent according to manufacturer instructions (Invitrogen Canada Inc., Burlington, ON, Canada). Total RNA was quantified using the Quant-iT™ RiboGreen^®^ RNA Assay Kit (Molecular Probes, Eugene, OR, USA), and quality was verified (RNA quality index (RQI)/RNA integrity number greater than 7.5) using Experion™ RNA StdSens analysis kits (Bio-Rad Laboratories Ltd., Mississauga, ON, Canada).

### 2.6. Gene Expression Analyses

Complementary DNA (cDNA) synthesis was performed using MultiScribe reverse transcriptase and random hexamers (Applied Biosystems, Mississauga, ON, Canada). Universal Probe Library design software (Roche Diagnostics, Laval, QC, Canada) was used for primer design (Table S1), and reaction efficiency (>90%) was verified using a cDNA dilution series. PCR reactions were performed in duplicate on 384-well plates using BrightGreen 2× qPCR MasterMix (Applied Biological Materials Inc. Richmond, BC, Canada) in a spectrofluorometric thermal cycler (Lightcycler 480, Roche Diagnostics) according to the following protocol: 3 min incubation at 95 °C followed by 50 cycles of denaturation at 95 °C, annealing at 60 °C, and elongation at 72 °C, each for 10 s. Product purity was verified by confirming a single peak for each analyte via melt curve analysis. Relative gene expression was calculated using β-actin as the reference gene according to the delta-delta Ct method [24].

### 2.7. Statistical Analyses

Sigma-Plot 12.5 (Systat Software Inc., San Jose, CA, USA) was used for all statistical analyses and generation of figures. Data were transformed to meet the requirements of normality and equal variance as warranted. Significant effects were determined by two-way ANOVA (factors *Ozone* (0, 0.8 ppm) and *Strain* (F344, LEW)) followed by the Holm–Sidak multiple comparison procedure (α = 0.05). Pearson correlation was conducted to test the relationships between lavage corticosterone and CBG.

## 3. Results

### 3.1. Effects of O_3_ on Glucocorticoid-Regulating Factors in the Lungs

**Figure 1:**
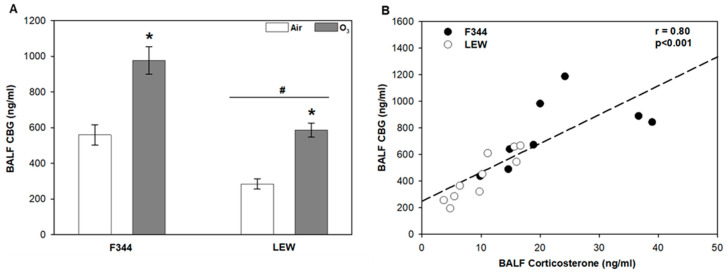
Strain comparison of lung lavage corticosteroid-binding globulin (CBG) after ozone inhalation. A. CBG levels in bronchoalveolar lavage fluid (BALF) in Fischer (F344) and Lewis (LEW) exposed to air or 0.8 ppm ozone (O_3_) for 4 h. * *Ozone* main effect, *p*  <  0.001; # *Strain* main effect, *p*  <  0.001; two-way analysis of variance (ANOVA) with Holm–Sidak post-hoc test; *n*  =  4–5 rats/strain/treatment. Data are expressed as mean ± SEM. B. Relationship between BALF corticosterone and CBG in F344 and LEW (Pearson correlation).

Levels of the high-specificity and -affinity corticosteroid-binding globulin (CBG) were elevated in F344 compared to LEW (*Strain* main effect, *p* < 0.001; Figure 1A). Exposure to O_3_ significantly increased lavage CBG (~1.7–2 fold) in both F344 and LEW, albeit with lower overall levels in LEW (*Ozone* main effect, *p* < 0.001). The profiles were similar to corticosterone profiles in plasma and lung lavage described previously [10,25]. Overall, there was a significant positive association between corticosterone and CBG recovered by bronchoalveolar lavage (r = 0.80; *p* < 0.001; Figure 1B).

**Figure 2:**
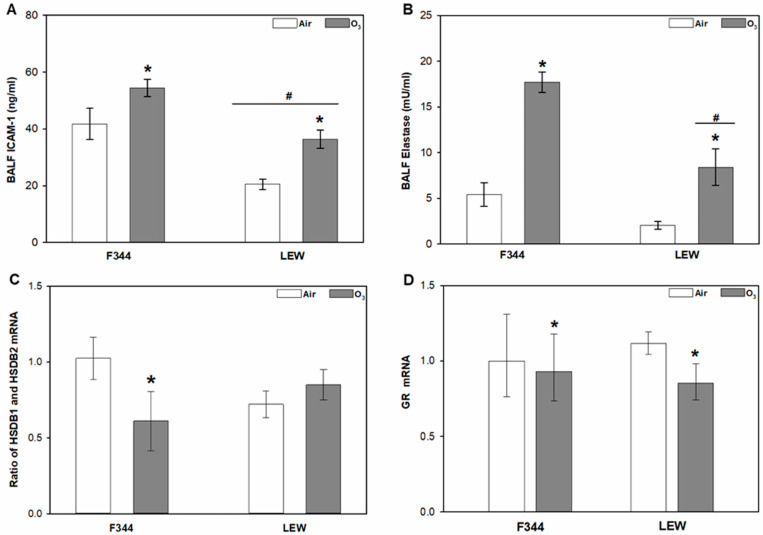
Glucocorticoid-regulating factors in the lungs in Fischer (F344) and Lewis (LEW) rats after exposure to air or 0.8 ppm ozone (O_3_) for 4 h. (**A**) Protein levels in bronchoalveolar lavage fluid (BALF) of intercellular adhesion molecule-1 (ICAM-1); *Ozone* (*p* = 0.001) and *Strain* (*p* < 0.001) main effects. (**B**) Elastase activity in BALF; *Ozone* × *Strain* interaction, *p* = 0.048. (**C**) 11β-Hydroxysteroid dehydrogenase 1 (HSDB1) and HSDB2 ratio (calculated from the mRNA abundance data); *Ozone* main effect, *p*  =  0.030. (**D**) Glucocorticoid receptor (GR) mRNA levels in lung tissue; *Ozone* main effect, *p*  =  0.01. * 0.8 vs. 0 ppm ozone within given strain (except for GR where an asterisk indicates the *Ozone* main effect across strains); # LEW vs. F344 within indicated exposure level; two-way ANOVA followed by Holm–Sidak post-hoc test; *n*  =  4–5 rats. For (**A**–**C**), data are expressed as mean ± SEM and for (**D**) data are expressed as geometric mean of fold-change relative to the F344 air control ± 95% confidence intervals.

In addition to binding of glucocorticoids, other factors controlling glucocorticoid entry and action in the lungs include regulation of lung permeability, glucocorticoid metabolism, and glucocorticoid receptors. Levels of ICAM-1, a factor regulating inflammatory response and lung permeability, were significantly higher in F344 compared to LEW, and were increased by O_3_ in both strains (*Strain* main effect, *p* < 0.001; *Ozone* main effect, *p* < 0.001; Figure 2A). Ozone exposure also increased total elastase activity in lavage collected from both F344 and LEW when compared to their air-exposed counterparts, with greater elastase activity observed in F344 compared to LEW (*Ozone* × *Strain* interactions; *p* = 0.048; Figure 2B). The mRNA ratio of the glucocorticoid metabolism factors HSDB1 and HSDB2 was reduced by approximately 40% by O_3_ in F344, an effect not observed in LEW (*Ozone* × *Strain* interaction; *p* = 0.03; Figure 2C). Overall, mRNA levels of the glucocorticoid receptor (GR) were decreased after O_3_ exposure (*Ozone* main effect, *p* < 0.01; Figure 2D), but no *Strain* effect was observed. No effect of *Ozone* or *Strain* was observed for mineralocorticoid receptor (MCR) gene expression (Figure S1).

### 3.2. O_3_-Dependent Inflammatory Signaling in the Lungs

**Figure 3:**
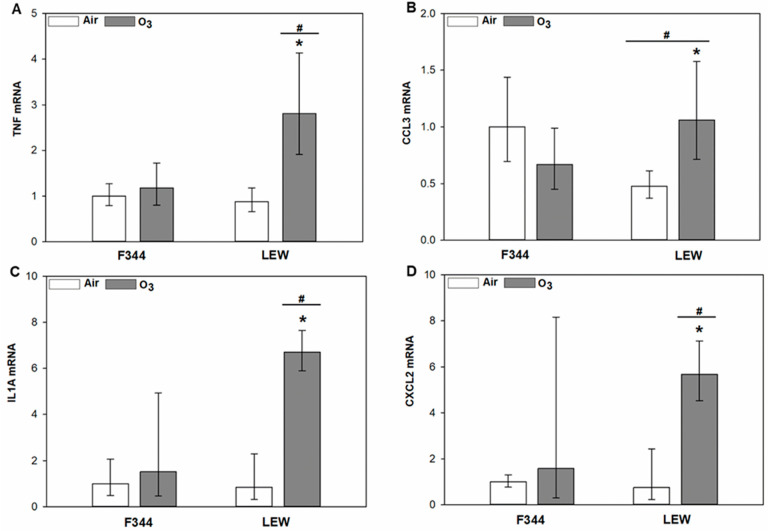
Expression of inflammatory genes in the lungs of Fischer (F344) and Lewis (LEW) rats after exposure to air or 0.8 ppm ozone (O_3_) for 4 h. Real-time PCR results are expressed as geometric mean of fold-change relative to the F344 air control ± 95% confidence intervals. (**A**). TNF. *Ozone* × *Strain* interaction, *p* < 0.001. (**B**). CCL3. *Ozone* × *Strain* interaction, *p* = 0.001. (**C**). IL1A. *Ozone* × *Strain* interaction, *p* = 0.01. (**D**). CXCL2. *Ozone* × *Strain* interaction, *p* = 0.006. * 0.8 vs. 0 ppm ozone within given strain and # LEW vs. F344 within indicated exposure level (two-way ANOVA followed by Holm–Sidak post-hoc test; *n*  =  4–5 rats).

Strain-dependent differences in the response of inflammatory genes to exposure were observed in lung tissue samples (Figure 3A–D). Compared to F344, the response of pro-inflammatory genes was significantly higher in LEW after O_3_. Significant *Ozone* × *Strain* interactions were observed for tumour necrosis factor (TNF; *p* < 0.001), C-C motif chemokine ligand 3 (CCL3; *p* = 0.001), interleukin-1α (IL1A; *p* = 0.01), and C-X-C motif chemokine ligand 2 (CXCL2; *p* = 0.006). Expression of inflammatory genes increased by 2.2–7.9 fold in O_3_ exposed LEW when compared to their air exposure counterparts.

## 4. Discussion

Immunomodulatory effects of endogenous glucocorticoids are well understood, and it has been demonstrated that impaired glucocorticoid release or signaling can modify lung inflammatory processes [16,26,27]. Since the ability of glucocorticoids to regulate inflammation is linked to their availability at sites of inflammation, factors that modify glucocorticoid availability in the lungs could impact air pollutant-induced lung inflammatory responses. We have previously demonstrated that exposure to O_3_ increases levels of the glucocorticoid corticosterone in the lungs of rat strains with distinct stress axis responsiveness, but that the profile of response differed by strain [25]. This prompted us to investigate how factors that modify glucocorticoid availability in the lungs differ across strains with contrasting stress axis responsiveness, and how exposure to an air pollutant may impact these factors. In the present study, we demonstrated that O_3_ exposure modified several factors that affect local glucocorticoid availability in the lungs; a subset of effects differed by strain. The data suggest that glucocorticoid regulatory factors may contribute to pulmonary effects of air pollutants, providing insight into a potential mechanism underlying differential sensitivity to pollutant exposure.

The major factors that affect glucocorticoid availability in the lungs include glucocorticoid-binding proteins, neutrophil elastase, and glucocorticoid metabolism enzymes HSDB1 and 2. Although it is suggested that only free glucocorticoids enter peripheral tissues [28], plasma glucocorticoid-binding proteins (CBG and albumin) are reported in the lungs, and their levels vary between individuals and are impacted by various diseases [12,28,29,30]. The ozone-dependent increase in CBG recovered in bronchoalveolar lavage fluid generally tracked levels of corticosterone, with higher levels observed in F344 compared to LEW. We have previously shown that acute exposure to 0.8 ppm O_3_ decreased total cell recovery by bronchoalveolar lavage in both strains immediately after exposure, consistent with increased production of adhesion factors; at the same time, we observed higher levels of lung injury markers in F344 compared to LEW [10]. In the present study, soluble ICAM-1, a marker of injury and lung permeability [31], was increased by exposure to O_3_ in both F344 and LEW, in line with the notion that glucocorticoid-binding proteins may enter from circulation after acute O_3_ exposure. The lungs are also a site of CBG synthesis [28,30], suggesting the potential for involvement of local production in regulating glucocorticoid activity.

It has been demonstrated that neutrophil elastase cleaves a reactive center loop of CBG, thereby enabling the local release of glucocorticoids at the site of inflammation [12,28]. In the present study, lavage elastase activity was significantly increased in F344 and LEW after O_3_ exposure, with more pronounced activity observed in F344. However, the substrate used was not specific to neutrophil or macrophage elastase activity; given the lack of neutrophils recovered by lung lavage at this early time point [25], it is possible that the present study measured macrophage elastase (MMP12) activity instead of neutrophil elastase. Although macrophage elastase (MMP-12) is reported to degrade extracellular matrix components including elastin and is implicated in tissue remodelling processes [32], there is no evidence that macrophage elastase cleaves CBG. Nevertheless, increased MMP12 activity may contribute to lung permeability that results in an influx of plasma glucocorticoid-binding proteins to the lungs and that in turn impact local glucocorticoid availability [33].

Regulation of other factors implicated in tissue-level control of glucocorticoid action may also be important. 11β-hydroxysteroid dehydrogenases (HSDB1 and 2) are considered intracellular regulators of tissue glucocorticoid action [14], as these enzymes control intracellular metabolism of endogenous glucocorticoids. It has been demonstrated that levels of these enzymes can vary with disease states, and considerable inter-individual variability exists in HSDB activity in humans [14,34]. In the present study, O_3_ exposure reduced the ratio of HSDB1/HSDB2 mRNA in the lungs of F344 but not LEW, suggesting strain-specific differences in the regulation of glucocorticoid metabolism in these animals. Glucocorticoid signalling in target tissues is, in turn, mediated primarily through binding to receptors; moreover, there is significant inter-individual variability in GR expression, and chronic impairment in GR function can lead to lung glucocorticoid resistance [35,36]. Here, O_3_ exposure downregulated lung GR expression in both F344 and LEW, consistent with known effects of glucocorticoid exposure [11,37]. The decrease in lung GR mRNA suggests that despite the strain differences in CBG and glucocorticoid metabolism factors, lung glucocorticoid levels in both rat strains reached a threshold sufficient to induce biological effects.

Differential regulation by air pollutants of factors important to the control of glucocorticoid may have physiological relevance. We have previously shown that pharmacological inhibition of the O_3_-dependent increase in corticosterone was associated with increased levels of inflammatory cytokines in the lungs [9]. In the present study, expression of several pro-inflammatory genes was increased by O_3_ in the lungs of LEW but not F344. These inter-strain differences may be due to the differences in both the levels of glucocorticoids and those factors that regulate their entry and activity. Indeed, it has been demonstrated that glucocorticoids at lower levels facilitate inflammatory responses, whereas at higher levels they dampen such responses [38]. Accordingly, the results are consistent with the notion that the higher glucocorticoid levels in F344 rats reduce lung inflammatory responses to O_3_ in F344, compared to less inhibition by the lower glucocorticoid levels in LEW. In a randomised double-blind crossover study, we recently showed that short-term exposure to diesel exhaust increased plasma cortisol levels, with the greatest effects seen in those with asthma or with risk alleles for antioxidant genes [7]. Investigation of inter-individual differences in glucocorticoid response and regulation in the lungs may provide insight into factors affecting the course and treatment of inflammatory lung diseases.

Our study provides evidence that local glucocorticoid-regulatory factors are affected by O_3_ exposure and, therefore, may play a role in the regulation of glucocorticoid signaling in the lungs following pollutant exposure. Furthermore, our results suggest that inter-individual differences in stress axis function may contribute to differential inflammatory responses following pollutant exposure. Given the important role played by glucocorticoids in the regulation of lung inflammatory responses [15,16,39], variability in glucocorticoid regulatory factors in lungs that subsequently modify glucocorticoid availability could contribute to differential susceptibility to air pollutants.

## Supplementary Materials

The following are available online at https://www.mdpi.com/article/10.3390/toxics9110290/s1, Figure S1: Expression of a glucocorticoid-regulating gene (mineralocorticoid receptor; MCR) in lung tissue of Fischer (F344) and Lewis (LEW) rats after exposure to air or 0.8 ppm ozone (O_3_) for 4 h. Real-time PCR results are expressed as geometric mean of fold-change relative to the F344 air control ±95% confidence intervals, Table S1: Gene-specific primer sequences for the quantitative real-time PCR used in this study.
