# Supplementary material for: Modulation by Ozone of Glucocorticoid-Regulating Factors in the Lungs in Relation to Stress Axis Reactivity
Source: Toxics. 2021 Nov 3;9(11):290. doi: 10.3390/toxics9110290 (PMC8622418; doi:10.3390/toxics9110290)
Supplement: Supplementary file 1 [file toxics-09-00290-s001.zip › toxics-1415808 SM.pdf]

toxics MDPI

Supplementary Materials: Modulation by ozone of glucocorticoid-regulating factors in the lungs in relation to stress axis reactivity

Jith Thomas and Errol M. Thomson

Table S1. Gene-specific primer sequences for the quantitative real-time PCR used in this study.

Gene		Primer	Accession Number
**ACTIN**	L	ctggctcctagcaccatga	NM_031144.2
	R	tagagccaccaatccacaca	
**IL1A**	L	aagagacggctaagtttcaatca	NM_017019.1
	R	tgatctgggttggatggtct	
**CXCL2**	L	gtccaaaagatactgaacaaaggc	NM_053647.1
	R	gttgaggtacaggagcccat	
**CCL3**	L	gcaccctctgttacctgctc	NM_013025.2
	R	taggagaagcagcaggcagt	
**TNF**	L	cgtagcccacgtcgtagc	NM_012675.3
	R	ggttgtctttgagatccatgc	
**GR**	L	aactggaataggtgccaagg	NM_012576.2
	R	ggtctcattccagggcttg	
**HSDB1**	L	cctcagctatgtggtcctgag	NM_017080.2
	R	cagaggttgggtcattttcc	
**HSDB2**	L	cgtcactcaaggggacgtat	NM_017081.1
	R	aggggtatggcatgtctcc	
**MCR**	L	caagctggcatgaacttagga	NM_013131.1
	R	tcctcgtggaggcctttt	

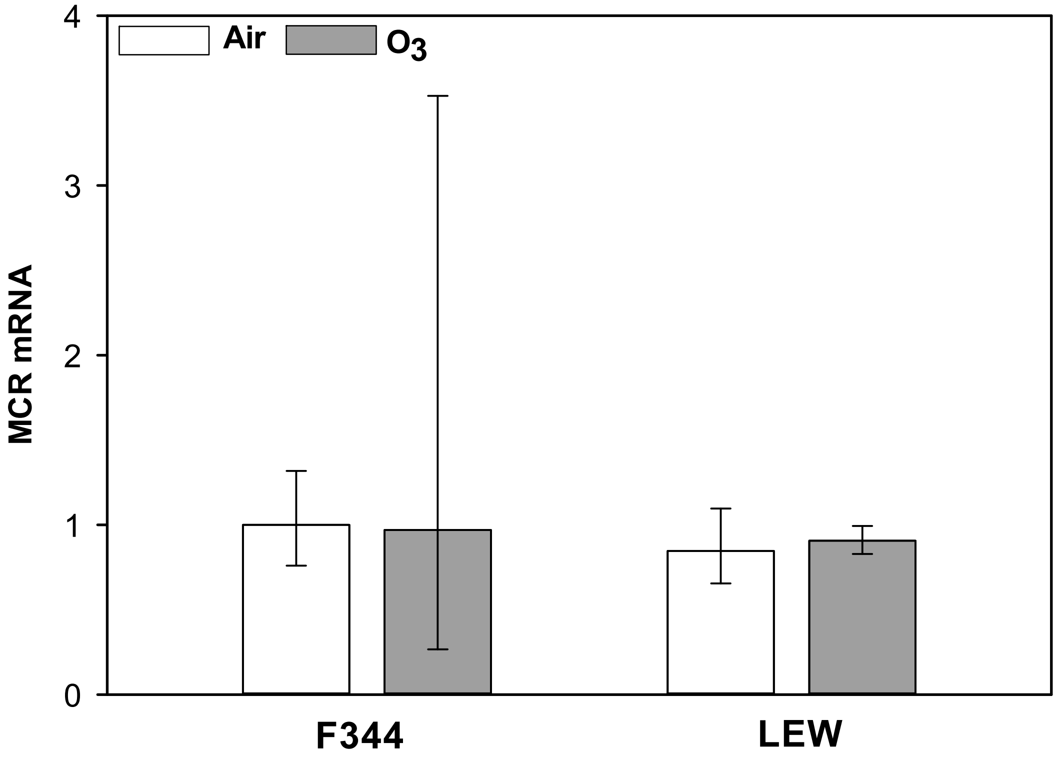

Figure S1. Expression of a glucocorticoid-regulating gene (mineralocorticoid receptor; MCR) in lung tissue of Fischer (F344) and Lewis (LEW) rats after exposure to air or 0.8 ppm ozone (O_3) for 4 h. Real-time PCR results are expressed as geometric mean of fold-change relative to the F344 air control ± 95% confidence intervals.
